# Fabrication of bio-engineered chitosan nanoformulations to inhibition of bacterial infection and to improve therapeutic potential of intestinal microflora, intestinal morphology, and immune response in infection induced rat model

**DOI:** 10.1080/10717544.2022.2081381

**Published:** 2022-06-29

**Authors:** Xiao Wan, Liu Liu, Lu Ding, Zhiqiang Zhu

**Affiliations:** a Department of General Surgery, Division of Life Science and Medicine, The First Affiliated Hospital of USTC, University of Science and Technology of China, Hefei, PR China; b School of Foreign Languages, West Anhui University, Lu’an, PR China

**Keywords:** Intestinal microflora, immune response, curcumin, MODE-K cells, antibacterial action

## Abstract

Overdosage of antibiotics used to prevent bacterial infections in the human and animal gastrointestinal tract would result in disturbing of intestinal barrier, significant misbalancing effects of intestinal microflora and persuading bacterial resistance. The main objective of the present investigation is to design and develop novel combinations of organic curcumin (Cur) and antimicrobial peptide (Amp) loaded chitosan nanoformulations (Cur/Amp@CS NPs) to improve significant effects on antibacterial action, immune response, intestine morphology, and intentional microflora. The antibacterial efficiency of the prepared nanoformulations was evaluated using *Escherichia coli* (*E. coli*) induced bacterial infections in GUT of Rat models. Further, we studied the cytocompatibility, inflammatory responses, α-diversity, intestinal morphology, and immune responses of treated nanoformulations in rat GUT models. The results indicated that Cur/Amp@CS NPs are greatly beneficial for intestinal microflora and could be a prodigious alternative of antibiotics.

## 1. Introduction

The gastrointestinal tract (GUT) of humans is a noteworthy and multifunctional organ, which is highly important for absorption of fluid and nutrient, osmoregulation and potentially act as a barrier to the exterior environment (Enoka et al., 2021). Generally, luminal contents have combinations of vital nutrients and essential micro-organisms including possibly harmful components of pathogens, pro-inflammatory factors, antigens, and toxins of synthetic and biological additives. Ingestion of harmful substances and uncontrolled passages across the intestinal epithelium tissues into the human circulatory system can cause severe tissue damages, infectious diseases, and inflammatory issues (Van den Mooter et al., 1993). Consequently, a protuberant function of the epithelium tissues is prominently needed to maintain discerning permeability functions, i.e. permitting uptake of necessary nutrients and ions while confining harmful components to reach circular system. Importantly, the intestinal barrier would be constituted of three types of layers: (i) extrinsic barrier produced of hydrated mucus gel with immune-efficient components, (ii) physical intrinsic barrier, containing epithelial cells associated at the apical membrane through tight junctions (labeled as TJ), and (iii) a primary immunological barrier, consisting of the GUT-associated lymphoid tissue (Abd El-Naby et al., 2020). The dysfunction of these intestinal barriers would mainly be the cause of acute stress, bacterial infections, viral infections, sub-optimal diet, and chemical exposures and obviously corresponded with an enhanced leakage of ions and particles into the lower layer epithelium of connective tissue, tissue damage, inflammatory issues lead to disease. Importantly, mechanisms of breakdown of the initial barrier can be associated to release of inflammatory cytokines, down-regulation of TJ protein expression levels and enhanced activity of intracellular kinases, plasma levels of stress hormone cortisol. Additionally, infected and impaired barrier functions are related with morphological variations including greater goblet cell abundance, enhanced immune cells infiltration and broadening of lamina propria (Ašmonaitė et al., 2018; Li et al., 2019; Yu et al., 2021).

Generally, microorganisms have a significant connection with human body mechanisms; specifically, diversity of GUT microbiome is vital for human health and microbial imbalances would create issues for microbiome of intestinal barrier and it has been demonstrated as inflammatory bowel diseases. The previous reports have widely demonstrated the substantial effects of microplastics (e.g. polystyrene) on the structural composition and functions of intestinal microflora in aquatic species and mammals (Elizalde-Velázquez et al., 2020; Zhang et al., 2021). The deviations on structural compositions in the intestinal barrier microbiome might have diverse effects on immune function. Some kinds of pathogenic bacterial have the capability to persuade the Treg cells differentiation and inflammatory process modulations by their anti-inflammatory and pro-inflammatory cytokines (Williams et al., 2015). Specifically, GUT microbes including *Bifidobacterium infantis* and *Firmicutes* have been explored to indorse the Treg cells induction and production of anti-inflammatory cytokines (Wang et al., 2012). As reported in studies, effective regulation of the ratio of Treg/Th17 cells could be helpful to maintain the immune responses of intestinal flora. Mainly, the acute infections caused by foreign substances and bacterial pathogens in GUT can persuade the appendicitis, cholecystitis, and peritonitis diseases (Li et al., 2020). Severe infections tempted by pathogenic bacteria would mainly cause life-threatening septicemia and blood infections, which disturbs millions of patients every year (Dang et al., 2021; Mazumder et al., 2021).

In recent years, over use and administration of antibiotics for infectious treatments severely causes adverse effects to human health, specially to intestinal system leads to acute diarrhea. Importantly, the world struggles severely by overuse of antibiotics and it would significantly cause kidney and liver dysfunction, which might lead to acute infections, and also produces antibiotic resistance (Binh & Toru, 2013; Xu et al., 2021). Orally taken broad-spectrum antibiotics would highly disturb the intestinal microflora, which affect bacterial pathogens along with probiotic existence in microbiome, and also kill intestinal epithelial cells (Scotia, 2010; Sochacki et al., 2011). In addition, antibiotic administrations to prenatal and postnatal infants may have high possibilities to modulate the composition of intestinal microflora, and induced obesity by increasing the effect of high-fat diet. The evidences from previous reports established that antibiotics exposures have long-term and severe negative effects on epithelial barrier function and intestinal homeostasis (Pei et al., 2019; Zhao et al., 2020). Hence, the new design and development of cell compatible and nontoxic alternatives to antibiotic is essentially required to treat antibiotic resistant bacterial pathogens. Antimicrobial peptides (AMPs) are positive charged and short peptides that can be discovered and isolated by different natural forms. The AMPs would have prominent ability to kill bacterial pathogens, which can be a capable alternative to drug-resistant antibiotics (Ramos et al., 2011; He et al., 2018). Among kind of AMPs, microcin J25 (MJ25) is a hopeful candidate and effective alternative of antibiotics. As previously reported, MJ25 AMP provides outstanding antimicrobial activity against predominantly gram-negative bacterial cells (G–) with low MICs in the nanomolar range. Importantly, MJ25 provide strong bactericidal efficiency against *Escherichia coli* (G–), *Salmonella* (G–), and *Shigella* (G–), meanwhile it did not show any toxicity against normal human cells *in vitro* and *in vivo* treatments (Yu et al., 2021). In addition, MJ25 have the prominent properties of low risk, greater stability, biocompatibility, expedient production method with excellent antimicrobial activity; hence, it is recommended as alternative candidate for drug-resistant antibiotics (Wang et al., 2020). Besides AMPs, natural anti-microbials (e.g. curcumin (Cur)) and chitosan-based antimicrobial materials would be a talented alternative for antibiotics. Significantly, chitosan-based antimicrobials in the form of nano- and micro-particles would be developed for drug carriers due to their low toxicity, greater compatibility with human cells and effective loading capability for hydrophilic drug molecules (Duan et al., 2010; George et al., 2019). Additionally, chitosan-based nanomaterials (CSNs) provide outstanding anti-microbial efficiency and broad-spectrum bactericidal activity against pathogenic bacteria in the absence of human cell toxicity and antibacterial resistance at working concentrations. Therefore, CSNs materials can be utilized as great candidate to apply for infectious diseases caused by anti-biotic resistant pathogens without adverse effects (Duan et al., 2010; Raj et al., 2018; Abd El-Naby et al., 2019).

Recently, traditional natural compounds have been primarily applied as antimicrobial agents and it would be a prodigious alternative for the antibiotics without adverse effects. Specifically, natural polyphenolic Cur molecule is an effective anti-microbial agent which is isolated from the curcuma longa Linn. (Zingiberaceae family) (O'Toole et al., 2016; Abdel-Hafez et al., 2018). Additionally, Cur is well-known for herbal medicine in Asian regions for the treatments of wound healing, sprains, and swellings produced by injuries. Importantly, nanoformulated CUR has been applied for anti-microbial, anti-inflammatory, anti-oxidant, and anti-tumor applications. The pharmacological and biomedical efficiency of the CUR formulations has been widely investigated and established by many researchers. The reported studies revealed that CUR can be extensively able to prevent inflammatory macrophages and monocytes released from TNF-α and IL-8 factors, meanwhile significantly influencing new skin regeneration and functional reconstruction processes by enabling the production of TGF-β signaling molecules (Basniwal et al., 2011; Karri et al., 2016). Although, CUR is well-known as hydrophobic polyphenol that has poor solubility nature in water, lower stability, and absorption behavior and rapid metabolism. In addition, that, concerning about the toxicity potential showed by high concentration of CUR, sustained release, solubility and stability of CUR could be modulated by impregnating it with chitosan molecules might be a favorable technique for application of drug delivery applications (Khan et al., 2016). In the present research report, we have designed and studied the therapeutic potential of AMP and CUR encapsulated CSNs materials using *in vitro* pathogenic models and *in vivo* rat models. The combinatorial effects of AMP/CUR with CSNs on rat intestinal microflora and immune system were evaluated after 28 days of administration, including microflora diversity and the wealth of Firmicutes, Bacteroidetes, and Actinobacteria in microflora. Further, structural/morphology characterizations and toxicity of fabricated material were investigated intricately by analytical and biological analysis methods, respectively.

## 2. Experimental

### 2.1. Materials

Curcumin powder and dichloromethane used for nano-Cur preparation were purchased from Sigma-Aldrich (St. Louis, MO). Chitosan (Mw 108 kDa (medium molecular weight); deacetylation degree 92%) and sodium tripolyphosphate (TPP) were obtained from Sigma-Aldrich (St. Louis, MO). AR acetic acid, dimethyl sulfoxide (DMSO), and MTT assay were purchased from Aladdin Chemicals Ltd. (Shanghai, PR China). The biological mediums including fetal bovine serum (FBS), Dulbecco’s modified Eagle medium (DMEM), and 1-ethyl-3-(3-dimethylaminopropyl) carbodiimide (EDC) were obtained from Sigma-Aldrich (St. Louis, MO).

### 2.2. Preparation of nano-curcumin

Primarily, 100 mg of Cur powder (0.27 mmol) was poured in dichloromethane (20 mL) under magnetic stirrer, and 2 mL of this prepared solution was gradually sprayed into the boiling water (40 mL) with a flow rate of 0.2 mL/min in 10 min under ultrasonic environment (100 W power; 30 kHz frequency). Then, the component was magnetically stirred for 30 min at 700 rpm and the obtained orange color solution concentrated under reduced pressure at 50 °C (Araki et al., 2020). The nano-Cur formation was confirmed by DLS (particle size) and TEM (morphology) analyses.

### 2.3. Fabrication of curcumin and AMP loaded CSNs particles

The preparation of chitosan microparticulate loaded with AMP and nano-Cur molecules under method of ionic gelation was established by Khan et al. (2016) and Taghipour-Sabzevar et al. (2020). Primarily, chitosan solution was prepared by dissolving chitosan powder 1% into the acetic acid (v/v; aqueous) and permitted to solubilize under magnetic stirrer for overnight at appropriate temperature. Meanwhile, aqueous solution of TPP was also prepared by dissolving 2 mg/mL concentration of TPP in the ultrapure deionized water. The prepared chitosan and TPP solutions were further filtered by using filter (0.45 µm pore size) before using for the reaction process. After that, different concentrations of AMP LL37 (200 µg) and nano-Cur were rapidly added to the chitosan solution under stirring process. To prepare CSNs particles, aqueous TPP solution was drop wise added to the chitosan mixture under continuous magnetic stirring process at 700 rpm for 1 h at atmospheric temperature (32 °C). The transparent solution was transferred to viscous nature, due to the forming of CSN particles. The obtained nanoformulations were separated by centrifugation (17,000 rpm) at 10 °C for 20 min and unreacted components were removed by further washing process by ultrapure distilled water and centrifugation methods. The final nanoparticulate product was stored at 4 °C for further analysis.

### 2.4. Evaluations of AMP encapsulation efficiency

The supernatant was stored and was used to investigate percentage of drug encapsulation efficiency. The percentage of encapsulation efficiency of CSN before and after loading of LL37 was measured at *λ*_max_=280 nm using Shimadzu UV-visible spectrophotometer (UV 2600, Kyoto, Japan). To investigate amount of AMP presented in each experiment, the corresponding blank CSNs particulate with the same morphology and structure without AMP were prepared and used as blank control versus UV absorbance of the supernatant. The existed amount of AMP in the supernatant was measured rendering to the obtained calibration curve value, which was drawn by obtaining the UV absorbance of identified concentrations of AMP. The measured concentrations were multiplied by the quantity of the supernatant to find out the amount of AMP presented in the supernatant. The encapsulation efficiency (EE%) and loading efficiency were calculated using the following equations:

$$\mathrm{EE}\%=(\frac{\left(\text{total amount of AMP used for preparing NPs}-\text{total amount of unloaded AMP within the supernatant}\right)}{\text{total amount of AMP used for preparing nanoparticles}})\;\times 100$$

$$\mathrm{LE}\%=(\frac{\left(\text{total amount of AMP used for preparing nanoparticles}-\text{total amount ofunloaded AMP within the supernatant}\right)}{\text{dry wright of nanoparticles}})\;\times 100$$

### 2.5. Characterization methods of nanoformulation

The average particles size and size distributions of the fabricated CSN particles loaded with CUR/AMP were evaluated under Beckman Coulter dynamic light scattering (LS230 Laser Diffraction PS analyzer, Nyon, Switzerland). The zeta potential value of the prepared nanomaterials was investigated under Delsa™ Beckman-Coulter Nano C instrument at 25 °C and pH 7.4 using SPB (10 mM). The chemical interactions and structural purity of the nanoformulations were evaluated by Fourier transform infrared spectroscopy (Nicolet FT-IR 6700; Thermo Fisher Scientific, Waltham, MA) analysis. The shape and morphological behavior of the AMP/CUR loaded CSN particles were investigated by field emission scanning electron microscope (FE-SEM; Hitachi S-4000; Hitachi High Technologies, Chatsworth, CA) and transmission electron microscopic (TEM; JEM 2000, JEOL Co., Tokyo, Japan) techniques.

### 2.6. *In vitro* cytocompatibility test

*In vitro* cell compatibility effects of prepared CSNs particulates with and without encapsulations of AMP/CUR were performed on human dermal fibroblast (HDF) cells. In brief, HDF cells were seeded in 96-well plates under DMEM growth medium containing FBS (10% (v/v)), penicillin (100 U/mL), and streptomycin (100 µg/mL) at suitable incubated settings (37 °C and 5% CO_2_). After cell confluence, about 75–90% were trypsinized using trypsin–EDTA (0.25%) solution and then cells at density 10^4^ were cultured on the 96-well plates. Then, different concentrations of bare nano-CUR, CSNs, and AMP/Cur-CSNs samples were distributed in freshly prepared DMEM medium and treated with seeded cells. Finally, quantitative cell survival rate was evaluated by MTT assay after incubation of 24 h (Li et al., 2019).

### 2.7. *In vitro* release analysis of curcumin and AMP

The prepared CSNs material loaded with AMP/CUR was dispersed in biological PBS medium (pH = 7.4) and then transported carefully to a 10 kDa dialysis bag. Directly, dialysis bag was moved in the PBS (50 mL) medium and was placed to stirrer at 120 rpm and 37 °C. During process, the dialysate medium (1 mL) was taken from beaker and replaced with same volume (1 mL) of fresh medium (PBS) at appropriate time durations. The releasing rate of AMP and CUR for CSNs was quantitatively measured using UV-visible spectrophotometer based on plotted CUR and AMP curve.

### 2.8. Animal experiments

#### 2.8.1. *In vivo* animal experimental design

The animal experimental procedure was reviewed and approved by the Institution Animal Care and Use Committee at University of Science and Technology of China, PR China. The experimental animals were housed and methodically handled in accordance with guidelines of National Institutes of Health for care and use of experimental animals. Primality, we created infected mice models by using *E. coli* gavage (1 × 10^9^ CFU/mL, 250 µL), and mice models administered with PBS saline only and without bacterial infection were considered as positive and negative controls, respectively. After that, 1.7 mg/kg of developed materials (bare Cur, AMP-CSNs, and Cur/AMP-CSNs) were administered to infected mice models. The feces of mice models were collected before and after sample administration at different durations (0, 12, 24, 48, and 72 h), and dispersed in PBS medium (1 mL). Then, we kept dispersed solution for shaking for two hours and centrifuged at 3500 rpm/min for 10 min to obtain the bacterial cells and counted the number of *E. coli* on plate. The blood, small intestines, and ceca were collected from sacrificed mice models by CO_2_ asphyxiation after 72 h for analysis.

At the same time, we allotted the mice models arbitrarily to experimental groups (4 mice/dose group), and administered them with prepared formulations (bare Cur, AMP-CSNs, and Cur/AMP-CSNs) with concentrations of 150, 1500, and 15,000 µg/kg by gavage during 28 days. All the treated mice were weighed every three days once during the experimental period. After 28-days of experiment, treated mice were starved for 12 h and sacrificed by CO_2_ asphyxiation, and collected their feces, blood, and small intestines for further analysis. Meanwhile, major organs of mice including livers, kidneys, spleens, lungs, and hearts were also collected to investigate the *in vivo* toxicity of the prepared materials.

#### 2.8.2. Intestinal morphology

The evaluation of morphological changes of collected parts (small intestines, liver, kidney, heart, and lung) was performed by using hematoxylin and eosin (H&E) staining as followed by previously reported methods (Yang et al., 2012; Ke et al., 2014; Dawood et al., 2020). The collected organ samples from mice were fixed by glutaraldehyde (2%) for 24 h, and then tissues were deposited on ethanol concentrations in ascending series and followed by treatment with dimethyl benzene. After that, tissues were dipped in paraffin for 3 h at 55 °C and embedded in paraffin at 4 °C. Finally, the H&E-stained images of tissues were recorded using microscope attached with digital camera system (Leica, Wetzlar, Germany).

#### 2.8.3. Analysis of immune response

The serum concentrations of immunoglobulin A (IgA), immunoglobulin M (IgM), immunoglobulin G (IgG), complement 3 (C3), and complement 4 (C4) were investigated and obtained the value quantitatively from collected serum by commercial kits as manufacturer’s instructions. Additionally, concentrations of tumor necrosis factor-α (TNF-α) and interleukin-6 (IL-6) were estimated using ELISA kits and all the samples were measured as manufacturer’s instructions.

#### 2.8.4. Bacterial inhibition and biofilm analysis

The populations of pathogenic *E. coli*, *Lactobacillus*, *Salmonella*, and *Bacillus bifidus* were evaluated from the collected contents of ceca, rectal, and colonic segments. Briefly, digesta (1 g) were collected from each treated sample and simultaneously diluted by sterile physiological saline solution 10-fold, resulting in dilutions ranging from 10^−1^ to 10^−6^. The *E. coli*, *Salmonella*, *Bifidobacteria*, and *Lactobacillus* were cultured in TPY medium and MacConkey agar, h and the culture plates were placed into an anaerobic incubation chamber at 37 °C for 24. Then, each bacterial dilution was systematically counted and bacterial survival rate was stated as log10 colony-forming units (CFUs) with mean of triplicated data. The measurement of bacteria in CFU by visual count of colonies would be a best replicate set from bacterial dilutions, which resulted in 30–300 colonies per plate.

### 2.9. Statistical analysis

The data were expressed using a one-way analysis of variance (ANOVA) test, and differences were statistically significant by Tukey’s test (SPSS 21.0 software, Chicago, IL). Data were expressed as the mean ± standard error of the mean (SEM). Values of *p*<.05 were considered as significant value. All the graphs were diagramed using GraphPad Prism 8.0 (La Jolla, CA).

## 3. Results and discussion

### 3.1. Physico-chemical, morphology, and structural analyze

**Figure 1.**
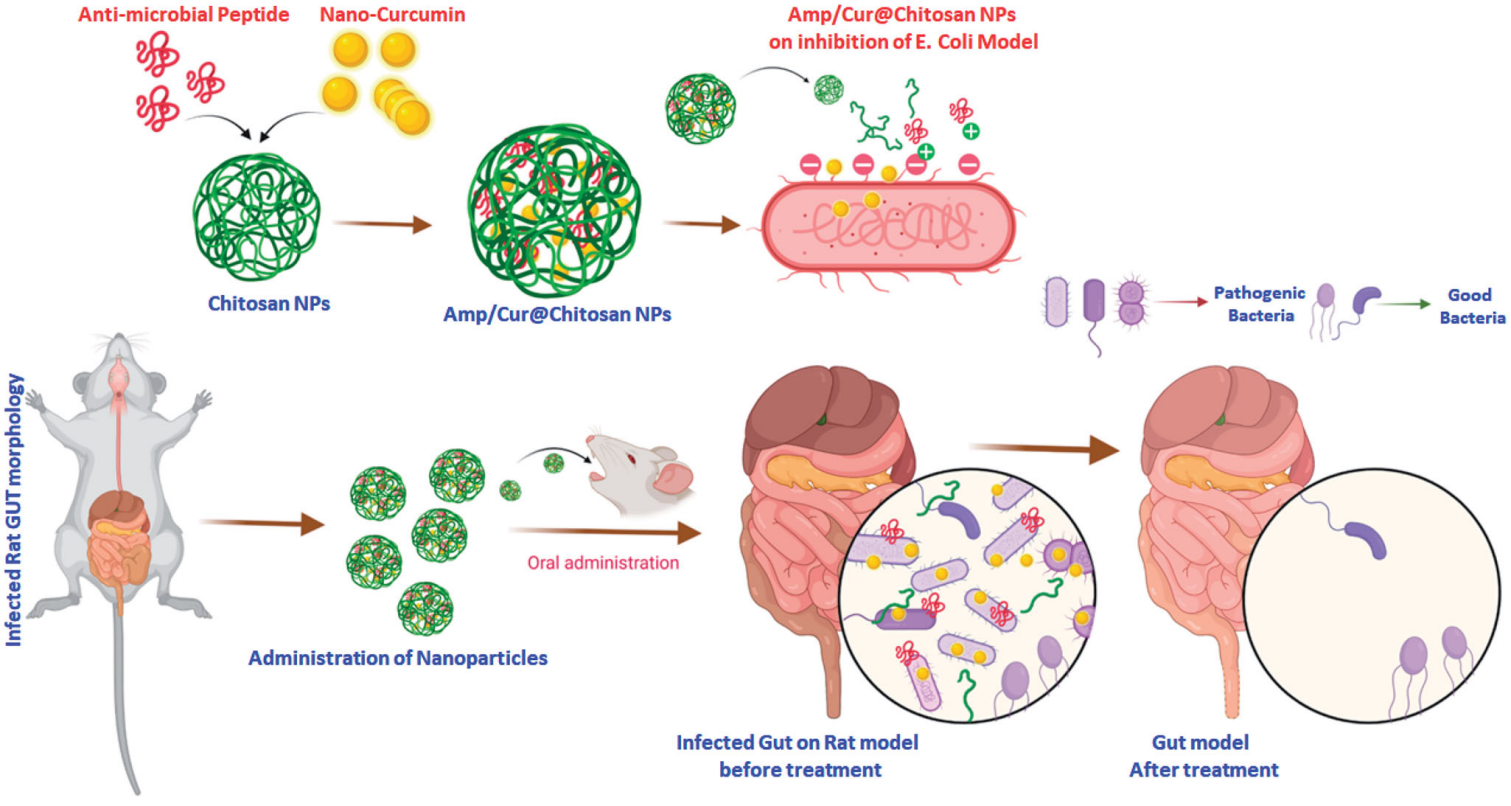
Schematic illustration of prepared nanoformulation encapsulated with curcumin and AMP components for anti-bacterial efficiency, intestinal microflora, and immune system.

**Figure 2.**
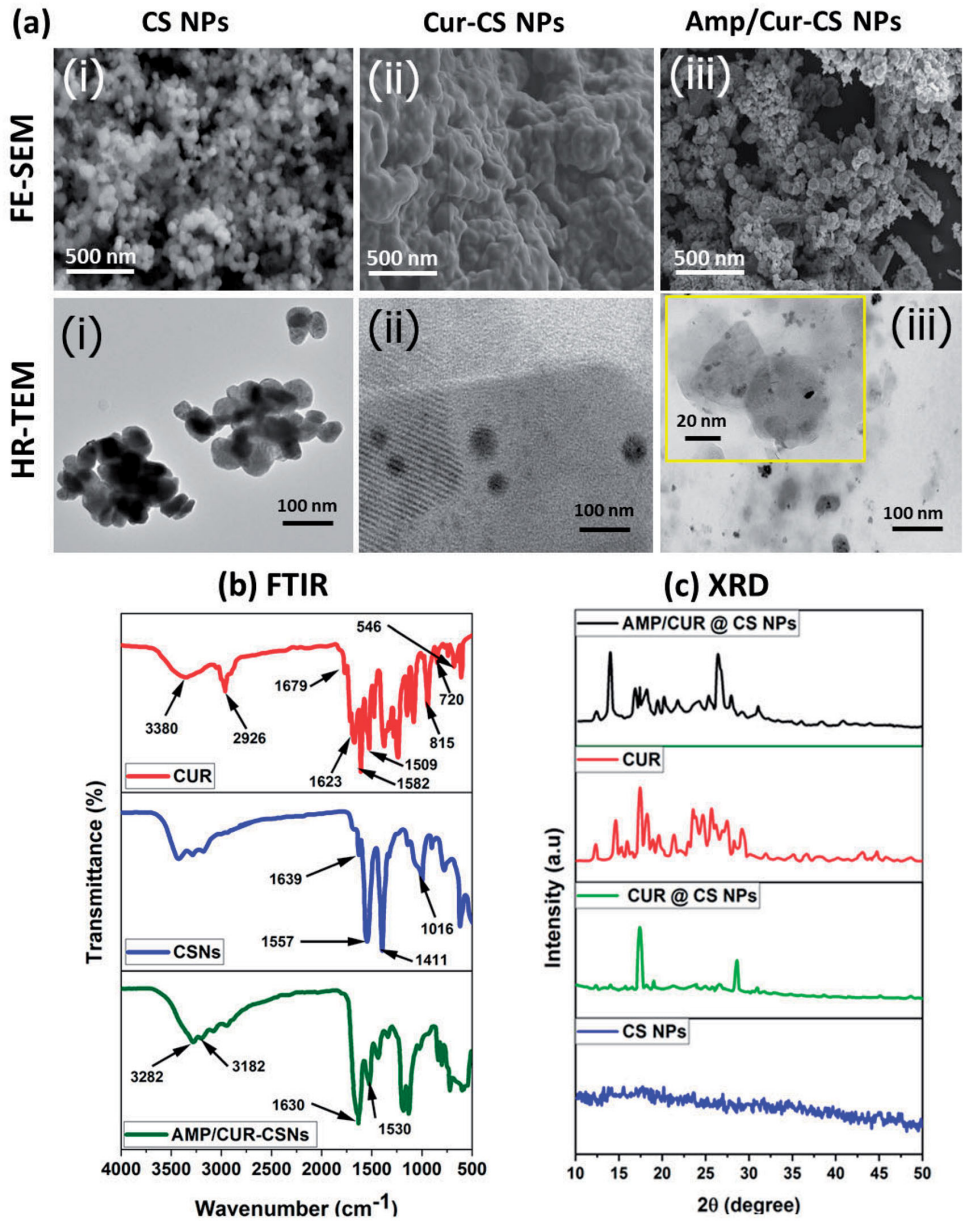
(a) SEM (top-order) and TEM (bottom-order) micrographs of the prepared (i) CS NPs, (ii) Cur-CS NPs, and (iii) Amp/Cur@CS NPs to exhibit the morphological structure; the FTIR (b) and XRD (c) analysis for chemical structural interactions and phase purity of the prepared CS NPs, Cur-CS NPs, and Amp/Cur@CS NPs.

In the present investigation, unique and highly compatible nanoformulation was prepared by interaction of positively charged chitosan molecules and negatively charged TPP molecules with optimized concentration (1:2 ratio mg/mL of CS and TPP) developed by Md. Azad Khan et al., to obtain effective size distribution, solubility, stability, morphology, and bioavailability. Additionally, the optimized CS nanoformulation loaded with Cur and AMP would have outstanding entrapment efficiency and also enhanced bioavailability of Cur molecules as reported in previous studies (Karri et al., 2016; O'Toole et al., 2016). The schematic representation of the present investigation has been presented in Figure 1 to exhibit the base idea of the study in microflora treatment. In the design and developing drug delivery carriers, morphology, particles size is an important factor and it has been suggested that nanocarrier size below 1 µm would be more beneficial to endure capillary distribution and uniform perfusion at the anticipated targeted site. The morphological properties of the nanoparticles were confirmed by SEM (upper order) and TEM (bottom order) analyses as exhibited in Figure 2(a). As shown in Figure 2(a) (upper order), SEM analysis confirmed that particles have formed spherical particles with smooth morphological nature for (iii) Amp/Cur@ CS NPs when compared to the (ii) Cur-CS NPs and (i) CS NPs. The surface structure of the nanoparticles was further confirmed by TEM analysis (Figure 2(a) (bottom order)), which specified that (iii) Cur/Amp@CS NPs and (ii) Cur-CS NPs have diverse spherical shape with dense structure, which was noticeably larger than that of average particles of bare (i) CS NPs. The mean particles size of the prepared nanoparticles as investigated by DLS analysis was found to be about 160–200 nm with narrow size distribution. The prepared CS NPs, Cur@CS NPs, and Amp/Cur@CS NPs exhibited that the average mean diameter of the nanoparticles is 160 ± 10 nm, 172 ± 14 nm, and 180 ± 12 nm, respectively, as shown in Table 1, with zeta potential value of 40 ± 1.2 mV, 37 ± 2.0 mV, and 46 ± 1.5 mV, respectively. The size distribution and surface charge of the nanoparticles are very significant factors for the drug delivered nanoparticles into the human body system. Though size of the particles could precisely administrate particle distributions into the body, zeta potential importantly regulates the stability of the particles and influences interactions of the nanoformulation and cell membrane. As reported previously, zeta potential value of the stable nanoformulations should be more than ±30 mV and can be highly suitable for the drug delivery system (Khan et al., 2020). The increasing particles size of CS NPs after cur/amp loading demonstrates the successful encapsulation of Cur/AMP molecules on the surface. Zhang et al. (1994) reported that increasing zeta potential value of the nanoformulation would confirm enhancing encapsulation efficiency of nanoparticles (Zhang et al., 1994). Importantly, binding of Cur molecules onto CS nanoparticles highly depends on pH and higher binding of Cur obtained at pH 5. In addition, maximum binding ability of Cur at lower pH is extra advantage for the Cur stability.

**Table 1.** The characterization measurements of prepared CS NPs, Cur@CS NPs, and Cur/Amp@CS NPs; drug loading efficiency (LE, %), encapsulation efficiency (EE, %), average particle diameter (size, nm), polydispersity index (PDI), and particle’s surface charge (zeta potential).

Formulation code	LE (%±SD)	EE (%±SD)	Size (nm ± SD)	PDI	Zeta potential (mV ± SD)
CS NPs	–	–	160 ± 10	0.223	40 ± 1.2
Cur@CS NPs	72.5 ± 3.2	76 ± 3.0	172 ± 14	0.171	37 ± 2.0
Cur/Amp@CS NPs	61.3 ± 2.5	73.5 ± 2.0	180 ± 12	0.175	46 ± 1.5

The Cur molecules and AMP loading and structural interactions of prepared chitosan NPs were further confirmed by the FTIR spectroscopic analysis as shown in Figure 2(b). The spectroscopic data showed the FTIR spectra of bare chitosan NPs, nano-Cur, and Cur/Amp loaded CS NPs. The spectrum of CS NPs exhibited the peaks at 1370, 1645, 2870, and 3200–3400 cm^−1^, which corresponded to the stretching of –C–O, bending vibration of NH group, stretching of aliphatic C–H group and stretching vibrations of –OH/–NH_2_ groups, respectively. In the spectrum of Cur, high intensity peaks appeared at 1520 cm^−1^, 1428 cm^−1^, and 850 cm^−1^, corresponding to stretching vibrations of C═C benzene ring, C–H olefinic bending vibrations, and C–O vibrations, respectively. The spectrum of Cur/Amp loaded CS NPs also displayed peak at 1635 cm^−1^ and 1080 cm^−1^, corresponding to the deformation of amino groups and -keto group presence due to the encapsulation of Cur molecules. Additionally, FTIR data also confirmed that successful drug loading and formation of chitosan nanoparticles cause interaction between the amino groups of chitosan and phosphor groups of TPP molecules.

Generally, Cur molecules incline to form crystalline nature when added to an aqueous medium due to its greater hydrophobic behavior. The crystalline nanoformulation of Cur molecules inside the chitosan matrix impedes the release of Cur and would be beneficial for controlled release of the drug delivery system. Therefore, the observation of XRD patterns was used to determine the crystalline properties of the prepared nanoformulations as exhibited in Figure 2(c). The XRD patterns of CS NPs and Cur loaded CS NPs showed broad peaks below 30° which established that semi-crystalline nature of the major component such as chitosan matrix. Specifically, Cur displayed multiplet appearance peaks at ranges between 2*ϴ*=2° to 70° indicating the well-crystalline nature of the nano-Cur molecules. The XRD pattern of bare CS NPs showed two identical crystalline peaks at 10° and 20.3° with reflections of (020) and (100), respectively, which demonstrated the semi-crystalline nature of bare chitosan polymer matrix. It was noted that the any respective peak of Cur and AMP was not observed in XRD diffractogram of Cur/Amp loaded CS NPs and confirmed that prepared nanoformulation is semi-crystalline nature, due to molecular level distribution and disordered crystalline properties of Cur and AMP molecules into the CS nanoparticles. In addition, disordered crystalline form of Cur molecules would highly be beneficial for controlled drug delivery system due to its constant diffusion from amorphous polymer matrix as reported in previous studies (Araki et al., 2020).

### 3.2. *In vitro* drug release

**Figure 3.**
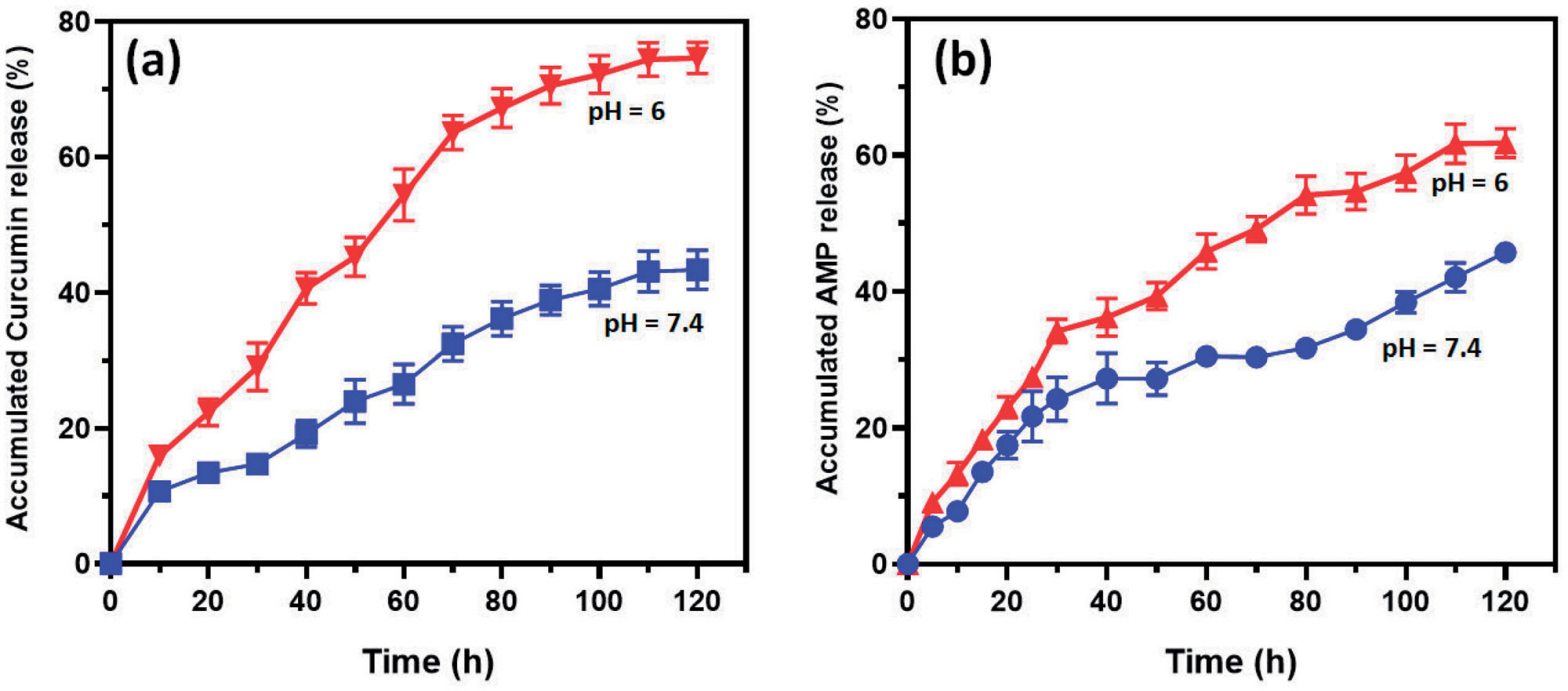
*In vitro* cumulative pH dependent release of (a) curcumin and (b) AMP from developed CS NPs in the medium of PBS at different pH conditions (6 and 7.4). The observed results are expressed as the mean of SE value (±) from three replicated experiments. Statistical analyses were performed by one-way ANOVA test using GraphPad Prism (8.0) (La Jolla, CA). *p*<.05 for PBS pH 6 and PBS pH 7.4 and differences were statistically significant.

The drug release profiles of Cur and AMP molecules from chitosan nanoformulation were observed by direct dispersion technique under different ranges at pH 5 and 7.4 (Figure 3). The release pattern of Cur (Figure 3(a)) and AMP (Figure 3(b)) established that drug molecules have burst release rate in the first four hours and followed by achieved sustained releasing rate from chitosan when increasing incubation time till 120 h and attained about 85% of Cur and 65% of AMP, respectively, released at that end duration. The initial burst release happened due to the reasons of some drug molecules entrapment on the surface of chitosan and higher dissolution rate of chitosan polymeric surface. Besides, we noted that the Cur and AMP molecules were quicker at lower pH (<6) conditions when compared to the higher pH (>7) because chitosan polymeric surface would get easy swelled at acidic environment due to the effective protonation reaction of amine groups presented in chitosan molecule. The determined *in vitro* release profiles of Cur and AMP clearly established that chitosan NPs have significantly provided sustained and prolonged release rate at pH conditions of 5.0 and 7.4.

### 3.3. *In vitro* compatibility

**Figure 4.**
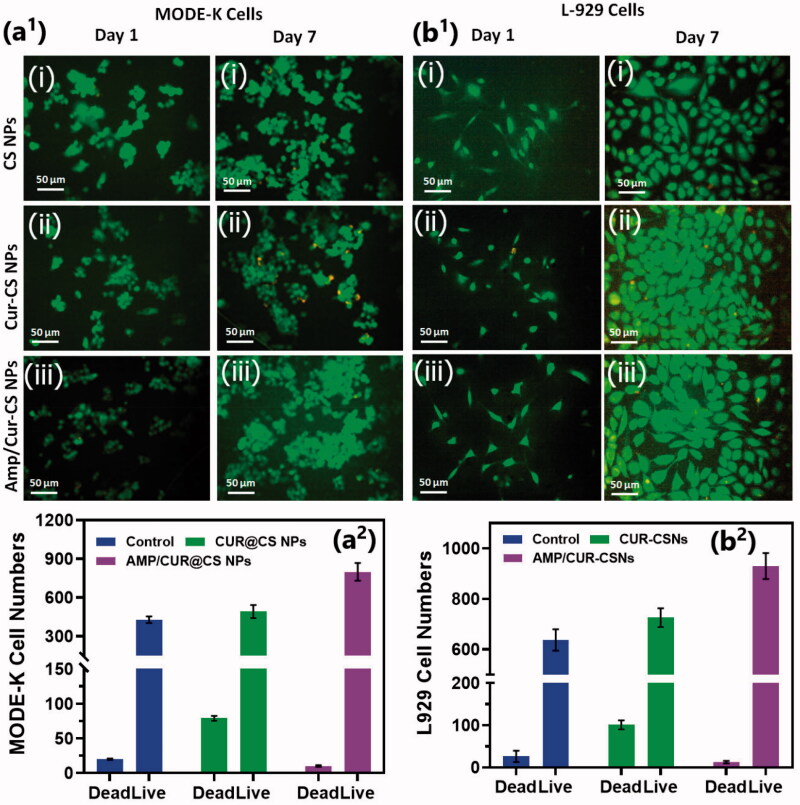
Fluorescence live/dead images of MODE-K cells (a^1^) and L929 (b^1^) were presented for different days of culture (day 1 and day 7) treated with prepared samples of (i) CS NPs, (ii) Cur-CS NPs, and (iii) Amp/Cur@CS NPs. Live cells (green) and dead cell (orange) were stained with calcein AM and PI, respectively, the cells were photographed by fluorescence microscope; quantitative measurement of live/dead cell numbers of MODE-K (a^2^) and L929 cells (b^2^) was exhibited to show efficiency of prepared (i) CS NPs, (ii) Cur-CS NPs, and (iii) Amp/Cur@CS NPs samples on cell proliferations.

We investigated the cytocompatibility of the developed CS nanoformulations loaded with Cur and AMP using MTT assay and Live-Dead staining kit on intestinal epithelial cells (MODE-K) and human fibroblast (L929) cells as shown in Figure 4. The observations of Live/Dead assay using confocal microscope established that live cell numbers of (iii) Cur/Amp@CS NPs and (ii) Cur@CS NPs were 20% and 15%, respectively, higher than (i) CS NPs, which indicated that addition of Cur and AMP molecules would provide more compatibility and survival ability of MODE-K (Figure 4(a^1^)) and L29 cells (Figure 4(b^1^)). We quantitatively measured the cell survival rate of MODE-K and L29 cells as exhibited in Figure 4(a^2^) and Figure 4b^2^, respectively, after they were cultured and treated with prepared nanoformulations of (i) CS NPs, (ii) Cur@CS NPs, and (iii) Cur/Amp@CS NPs samples and PBS medium used as control by observing at OD450 under spectrophotometrically method. We have investigated the Cur@CS NPs and Cur/Amp@CS NPs by using different concentrations (0, 63, 125, 250, 500, and 1000 µg/mL) on MODE-K cells to know optimized concentration, which confirms that about 500 µg/mL cell has no toxicity as shown in Figure SI 1. The observed results demonstrated that cell survival percentage of Cur/Amp@CS NPs was significantly higher than that of other sample including control.

### 3.4. Anti-bacterial efficacy

**Figure 5.**
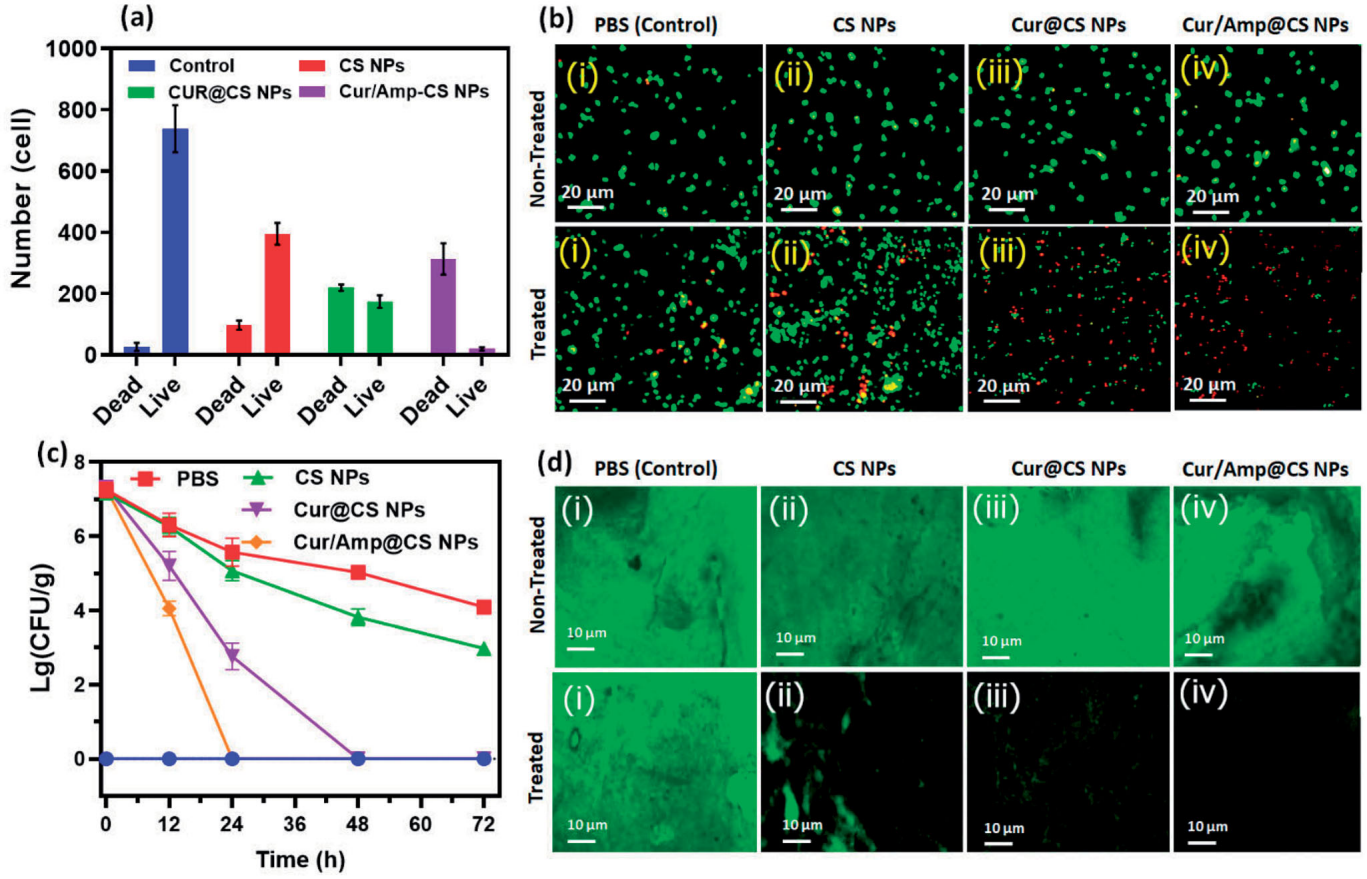
Antibacterial efficiency of prepared (i) control (PBS), (ii) CS NPs, (iii) Cur@CS NPs, and (iv) Amp/Cur@CS NPs was observed against *E. coli*; (a) quantitative value of live/dead ratio of *E. coli* and (b) live/dead fluorescent assay after treated with samples of (i) control (PBS), (ii) CS NPs, (iii) Cur@CS NPs, and (iv) Amp/Cur@CS NPs (scale = 20 µm) after 24 h. (c) The counting of colony forming unit (CFU) after treatment of different samples after 24 hours and (d) biofilm inhibition images of *E. coli* after 24 treated with samples of (i) control (PBS), (ii) CS NPs, (iii) Cur@CS NPs, and (iv) Amp/Cur@CS NPs (scale = 10 µm).

**Figure 6.**
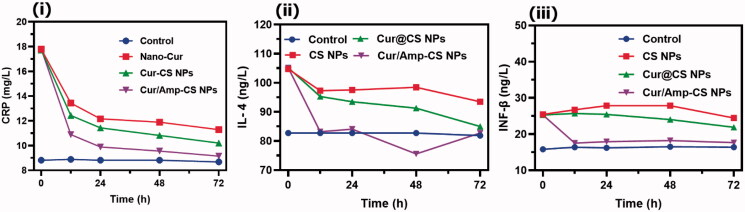
The quantitative observations of (i) CRP in treated animal serum to indicate the level of inflammation occurred and markers of (ii) IL-4 and (iii) INF-β which specified the inflammatory cytokine in the serum after treatment of control, CS NPs, Cur@CS NPs, and Amp/Cur@CS NPs samples.

The anti-bacterial therapeutic potential of the prepared nanoformulations (i) control (PBS), (ii) CS NPs, (iii) Cur@CS NPs, and (iv) Cur/Amp@CS NPs was evaluated using feces of infected mice by *E. coli* at dose of 1700 µg/kg, which has used LFX as clinical optimized dosage (Figure 5). The quantitative and qualitative live/dead assay of *E. coli* bacterial cells was investigated using AO/EB dual staining for effectiveness of cell live/death numbers (Figure 5(a,b)), which indicated that Cur/Amp@CS NPs have greater effectiveness than Cur@CS NPs and control groups. We obtained bacterial cells from feces of mice models after they were treated with specified nanoformulations individually at different time durations (0, 12, 24, and 48 h). The obtained results showed bacterial cell numbers of PBS treated group were 1.20 × 10^6^ CFU/g before treatment to mice groups and control group would be absence treatment of *E. coli* cells (Figure 5(c)). The number of bacterial cells was significantly decreased to 2.50 × 10^5^, 3.24 × 10^4^, and 2.9 × 10^4^ CFU/g for CS NPs, Cur@CS NPs, and Cur/Amp@CS NPs, respectively, after 12 h of infection. After 24 h of treatment, there were no bacterial cells treated with Cur/Amp@CS NPs and bacterial cell numbers tremendously decreased for the samples of Cur@CS NPs and CS NPs, respectively. After 48 h of observation, there were no bacterial cells observed for Cur/Amp@CS NPs and Cur@CS NPs, demonstrating that prepared CS nanoformulation with Cur and AMP has provided outstanding antibacterial potential against gut *E. coli* infection. The effective *E. coli* biofilm inhibition has been observed for Cur/Amp@CS NPs and Cur@CS NPs when compared to the CS NPs and control groups as shown in Figure 5(d). This research investigation confirmed that combined effects of Cur and AMP molecules would be more benefited than antibiotic bacterial action. In addition, we have also measured the amount of IL-4 (Figure 6(i)), CRP (Figure 6(ii)), and INF-β (Figure 6(iii)) markers from serum using ELISA kits to investigate the inflammation response in mice at different time durations. The results displayed that concentration of these markers significantly reduced for Cur/Amp@CS NPs and Cur@CS NPs treated samples after 12 h when compared to the CS NPs and control group.

### 3.5. Intestinal morphology

**Figure 7.**
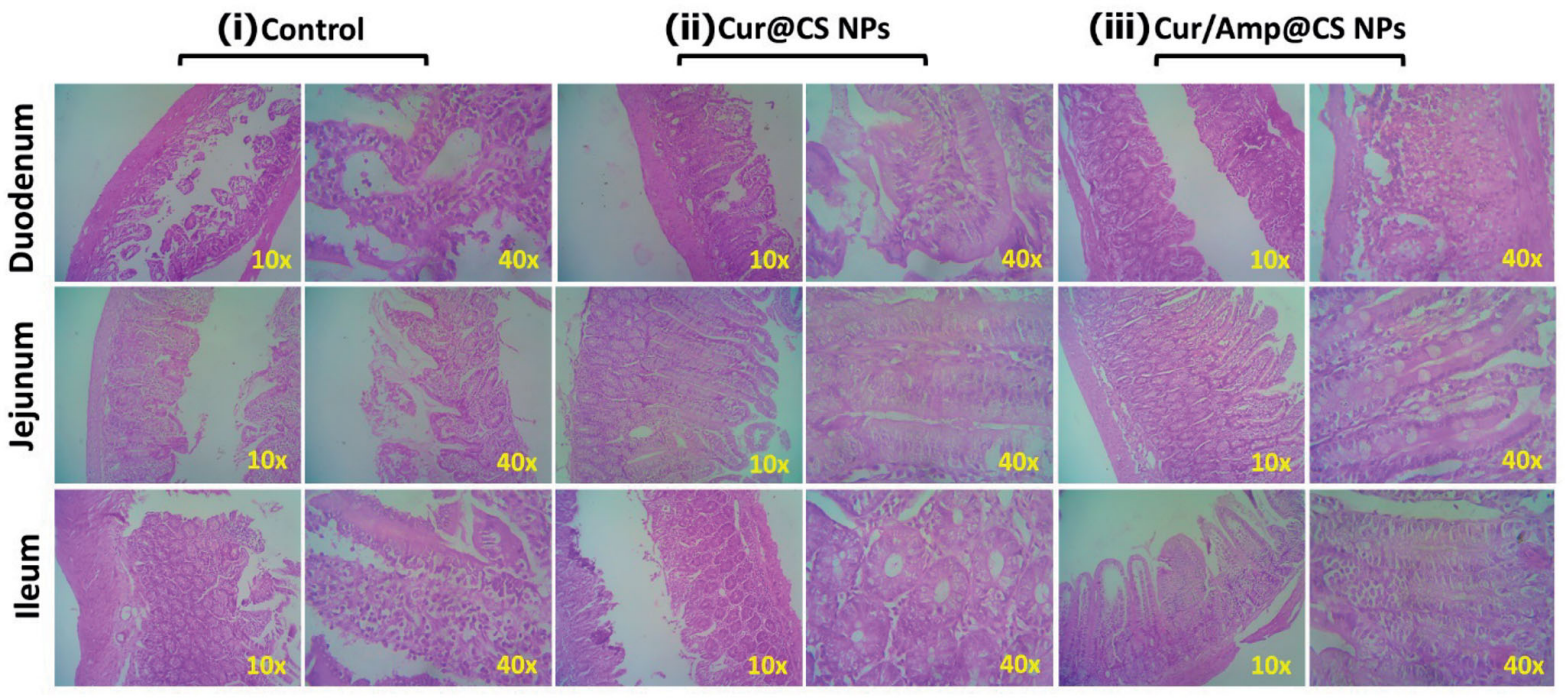
Histological images (HE staining) of *in vivo* intestinal morphology of different sections such as duodenum, jejunum, and ileum tissues after treated with (i) control, (ii) Cur@CS NPs, and (iii) Amp/Cur@CS NPs (magnifications ×10 and ×40).

The *in vivo* therapeutic potential of prepared nanoformulations was qualitatively investigated by histopathological observations of cross-sectioned rat GUT intestinal morphology after 28-days of administration as exhibited (Figure 7). The histopathological observations of Cur@CS NPs and control show pathological damages to villi in the ileum, duodenum, and jejunum. At the same time, Cur/Amp@CS NPs administrated models exhibited improved villous height and crypt depth of ileum, duodenum, and jejunum. The enhancement ratio of V and C in sections of duodenum and jejunum was measured in Cur/Amp@CS NPs with comparison of Cur@CS NPs. Generally, intestinal mucous membrane forms an epithelial cell constructed monolayer, would mainly contribute to the important intestinal functions of digestion, nutrients absorption and preventing disturbances of pathogens and toxic substances from intake foods. Commonly, digestive related syndromes and microbes’ infections could create intestinal dysfunction and impaired epithelial function significantly affect villus height, immune homeostasis, unbalancing absorptive-secretory electrolytes, enhanced inflammatory response, and upsetting barrier functions would lead to diarrhea. Our observations demonstrated that prepared nanoformulations Cur/Amp@CS NPs and Cur@CS NPs have significantly protected intestinal morphology (Figure 7) by upsurging the villus height, which was greatly consistent with previously proved results (Chen & Zhou, 2018; Pei et al., 2019).

### 3.6. Diversity of intestinal microflora

**Figure 8.**
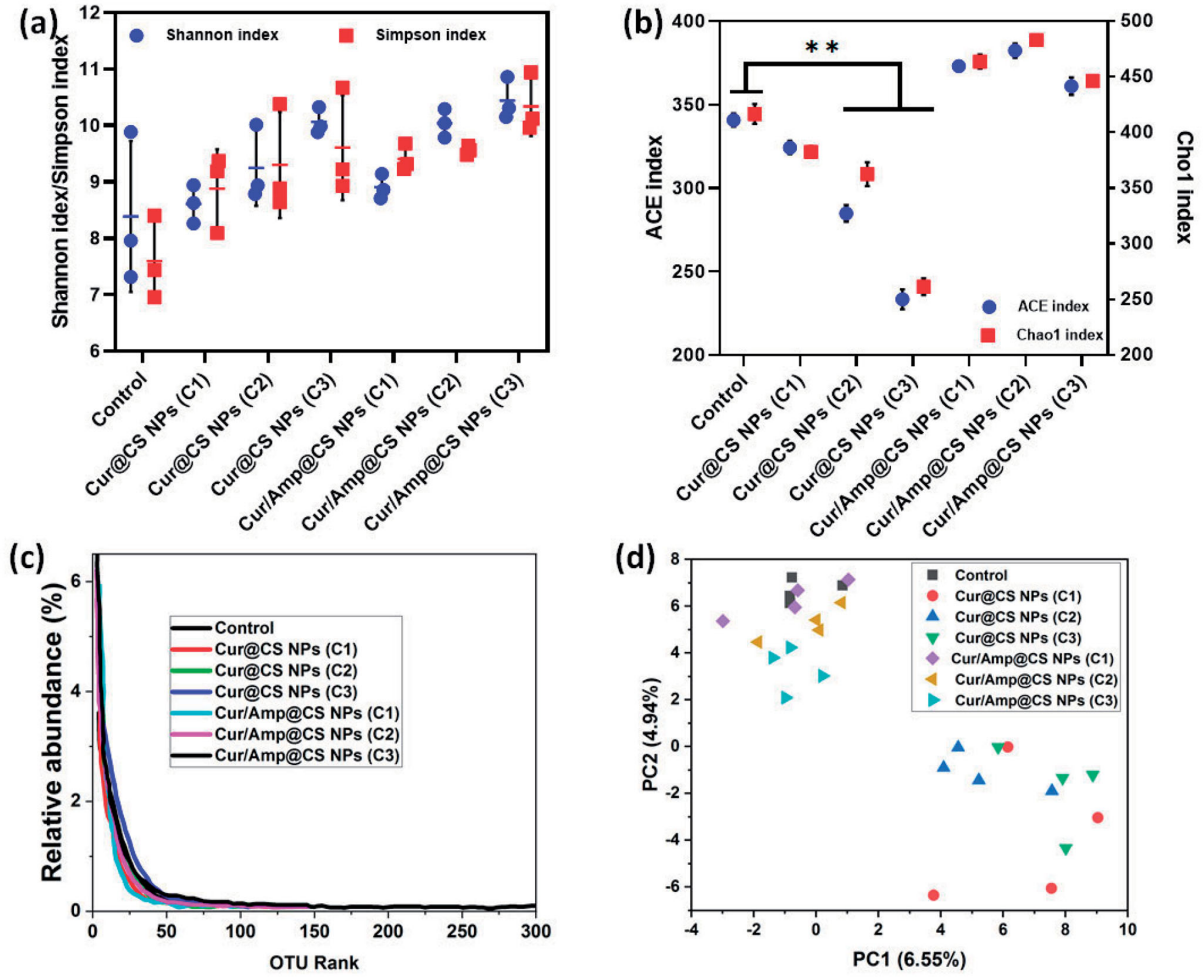
Observations of α-diversity of intestinal microflora in rat after administration of prepared samples for 28 days; (a) index values of Shannon and Simpson after treatment with different sample groups, which indicates prepared formulations have higher evenness; (b) index values of ACE and Chao1 and (c) OUT relative abundance after treatment, greater index values specify more richness of administered nanoformulations and (d) PCA ordination plot of intestinal microflora by different treated groups.

We performed the investigations of Shannon and Simpson index measurements (Figure 8(a)) and ACE and Chao1 index analysis (Figure 8(b)) to observe α-diversity of intestinal microflora richness and evenness, respectively after 28-day oral administration of prepared nanoformulations as protocols of previous study (Li et al., 2019). The presence of lower α-diversity range would have negative effects on intestinal microflora including obesity induction, insulin resistance, chronic inflammation and disorders of lipid metabolism. As shown in Figure 8, the results of Cur/Amp@CS NPs sample treated mice samples exhibited greater diversity values as higher rank abundance of OTU in intestinal microflora when compared to the Cur@CS NPs and control (Figure 8(c)). This observation has been confirmed that Cur/Amp@CS NPs would not disturb the microflora richness and evenness of treated mice after 28-days. The index values of Shannon/Simpson (Figure 8(a)) and ACE/Chao1 (Figure 8(b)) showed that no noticeable difference between Cur/Amp@CS NPs treated models and control groups. At the same time, Cur@CS NPs treated group have decreased richness of microflora when compared to the Cur/Amp@CS NPs treated models and control groups. Meanwhile, index values of ACE and Chao1 (Figure 8(b)) were decreased significantly from 328.5 to 260.4 and 332.4 to 240.3, respectively. Additionally, there is no difference in the index values of Shannon and Simpson (Figure 8(a)), revealing that the Cur@CS NPs have slightly reduced the richness of intestinal microflora. Further, we performed PCA analysis to investigate the efficiency differences between the microflora in different treated mice models (Figure 8(d)), which displays the respective distances between each points revealed the differences between the samples. The results of PCA observation on bacterial genera composition exhibited that Cur/Amp@CS NPs and control groups have differed microflora in mice models when compared to the Cur@CS NPs. As previous reports, long-term administration of antibiotic drug molecules would rapidly decrease the diversity of microflora, while Cur/Amp@CS NPs infrequently effect the diversity of microflora after a 28-day administration (Li et al., 2007, 2019).

**Figure 9.**
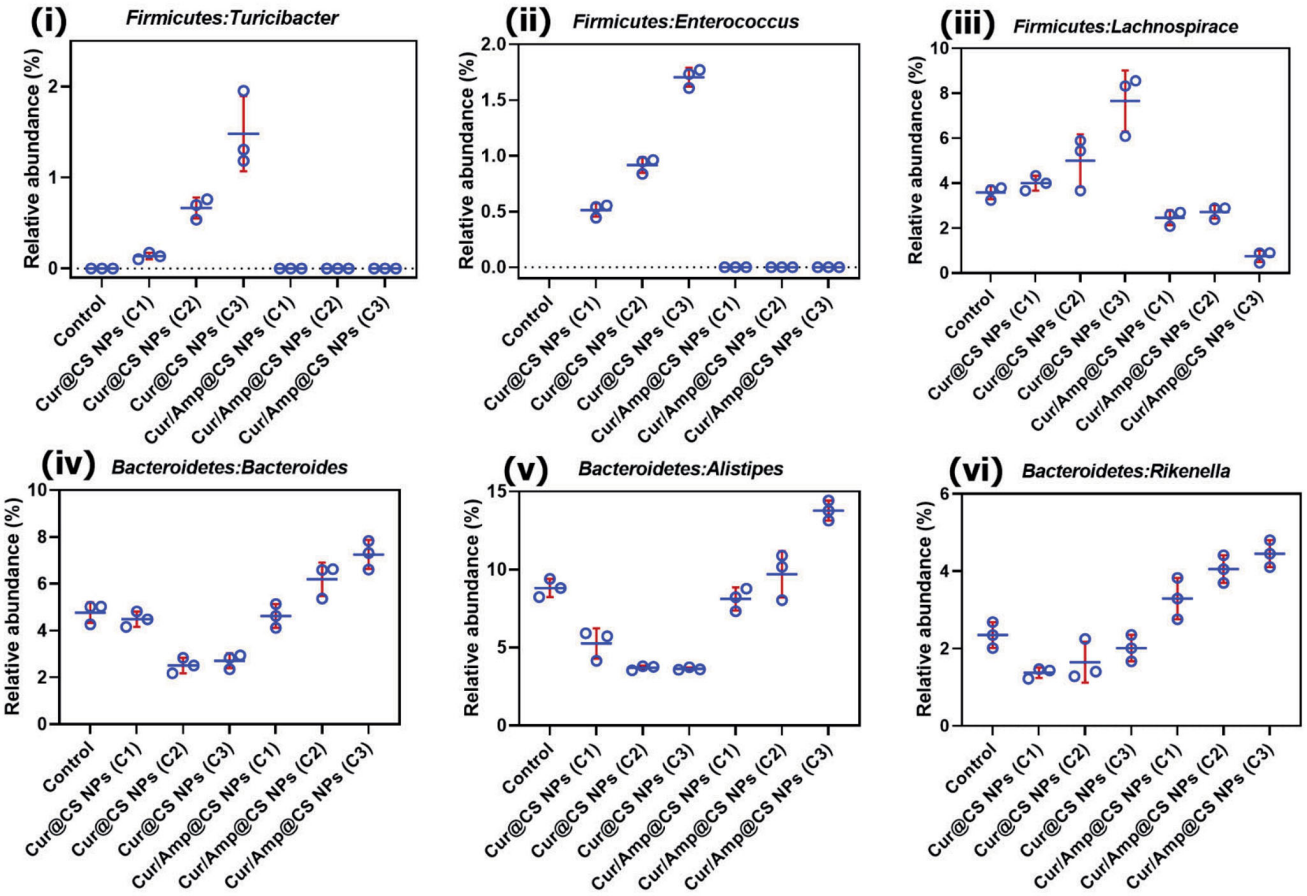
Quantitative values of relative abundance (RA) of genera in microflora; (i) Firmicutes: Turicibacter, (ii) Firmicutes: Enterococcus, (iii) Firmicutes: Lachnospiraceae, (iv) Bacteroidetes: Bacteroides, (v) Bacteroidetes: Alistipes, and (vi) Bacteroidetes: Rikenella species after 28-day administration of prepared Cur@CS NPs and Amp/Cur@CS NPs at different concentrations in rat models.

We performed the Firmicutes/Bacteroidetes (F/B) potential of intentional microflora after 28-day administration of prepared nanoformulations in rat as shown in Fig. SI2. The results exhibited that relative abundance (RA) F/B in microflora of rats was significantly changed after treatment of 28-days oral administration. The Cur/Amp@CS NPs has decreased the F/B value when compared to the Cur@CS NPs, which slightly have similar value to control group. The F/B values for Cur/Amp@CS NPs and Cur@CS NPs were 0.32 and 0.45 after 28-days administration. Generally, increasing value of Firmicutes confirms that occurrence of bacterial infections in GUT, which proves that Cur/Amp@CS NPs would provide beneficial effects on microflora by regulating dysfunctional microflora. Mainly, prepared nanoformulations significantly managed the F/B value of intestinal microflora with bacterial infections, revealing that it has been highly benefited the microflora system in rat GUT. Importantly, we have analyzed the therapeutic potential of nanoformulations on the RA of genera in F/B (Fig. SI2). In addition, we studied the RA of *Turicibacter* (Figure 9(i)), *Enterococcus* (Figure 9(ii)), and *Lachnospiraceae* (Figure 9(iii)) presented in Firmicutes, because they would mainly cause inflammation responses in GUT. The results exhibited that no formation of *Turicibacter* and *Enterococcus* after administration of Cur/Amp@CS NPs material at 28-days and RA value of *Lachnospiraceae* has been decreased when compared to the Cur@CS NPs and control groups (Figure 9(a–c)). The observation established that decreasing value of these genera in intestinal microflora after treated Cur/Amp@CS NPs would regulate the inflammation in rat intestine. As displayed in Figure 9(d–f), RA values of *Bacteroides* (Figure 9(iv)), *Alistipes* (Figure 9(v)), and *Rikenella* (Figure 9(vi)) in *Bacteroidetes* were studied after administration of 28-days, because they could obviously influence the metabolism of host. Normally, reducing occurrence of *Bacteroides*, *Rikenella*, and *Alistipes* in *Bacteroidetes* is strongly connected with various metabolic diseases including diabetes and obesity. The RA values of *Bacteroides* (Figure 9(iv)), *Alistipes* (Figure 9(v)), and *Rikenella* (Figure 9(vi)) have been increased to 6.55%, 13.5%, and 4.2%, respectively, after administration of Cur/Amp@CS NPs, which is greater when compared to the Cur/CS NPs and control groups. The results of distinctive genera in *Firmicutes* and *Bacteroidetes* were greatly consistent *(F/B)* values as shown Figure 9.

Importantly, we performed the RA values of *Verrucomicrobia* (i.e. *Akkermansia*) and *Actinobacteria* (i.e. *Bifidobacterium*) because these can be greatly regulating the substantial intestinal mechanisms of microbial homeostasis, inhibition of harmful bacterial pathogens, and significantly endorse the balancing atmosphere of intestinal microflora (Figure SI 3). As clinical reports, RA optimal values of *Akkermansia* and *Bifidobacterium* should be 0–3% and 0–10%, respectively, in intestinal microflora. The observed results indicated that RA values of *Akkermansia* and *Bifidobacterium* have been significantly augmented with administration of Cur/Amp@CS NPs and Cur@CS NPs. The RA of *Akkermansia* was 0.52% and 1.75% for Cur@CS NPs and Cur/Amp@CS NPs, respectively. The RA values of Bifidobacterium was 1.05% and 1.80% for Cur@CS NPs and Cur/Amp@CS NPs, respectively, as exhibited in Fig SI 3. The observations demonstrated that RA of *Akkermansia* and *Bifidobacterium* was significantly higher when treated with prepared nanoformulations, signifying that developed materials could be more beneficial by oral administration (Li et al., 2019).

### 3.7. Analysis of immune response

**Figure 10.**
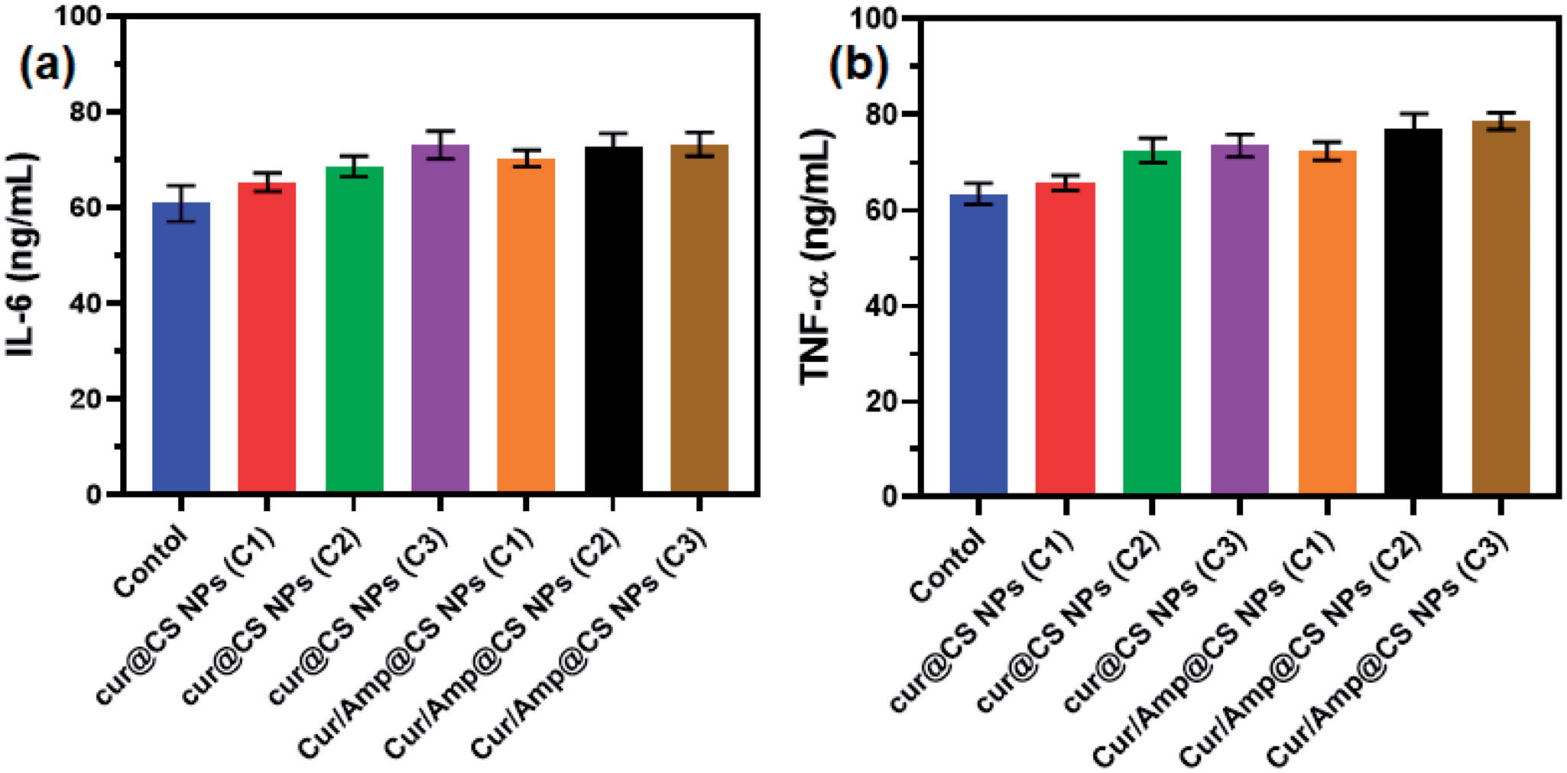
Quantitative measurement results of immune related gene expressions of (a) IL-6 and (b) TNF-α after treatment of prepared nanoformulations (control, Cur@CS NPs, and Cur/Amp@CS NPs).

The immune response efficiency of the prepared nanoformulations was performed on concentrations of cytokines and serum immunity indices of treated Rat models as exhibited in Figure 10 and Table 2. The blood concentration of IgA was significantly augmented with the Cur/Amp@CS NPs and Cur@CS NPs administrated groups when compared to the control group. In contrast, concentration of IgM was reduced and IgG has no difference with administration of different concentrations of Cur/Amp@CS NPs and Cur@CS NPs as shown in Table 2. Additionally, we performed the analysis for serum concentration of cytokine genes (IL-6 and TNF-α) as exhibited in Figure 10. The concentrations of IL-6 (Figure 10(a)) and TNF-α (Figure 10(b)) were significantly persuaded after administration of Cur/Amp@CS NPs and Cur@CS NPs when compared to the control group. As reports of previous studies, nanoparticles could have abilities to directly stimulate gut mucosa associated immune responses, meanwhile indirectly influencing the immune response mechanisms by modulating adherent bacterial populations (Yang et al., 2012; Qiao et al., 2019). Furthermore, it was proved that immune cells of intestinal mucosal tissues might have effective uptake larger size nanoparticles. The results established that administration of prepared nanoformulations exhibited have effectively down-regulated effects on inflammatory related genes (IL-6 and TNF-α). It was reported that, nanoparticles size also could be influencing factor to induce gene expressions and Cur/Amp@CS NPs have revealed up-regulations of gene expressions. These results demonstrated that gene expressions observed in the present investigations would be causes of components as well as nanoparticulate sizes (Rajchakit & Sarojini, 2017; Khan et al., 2020).

**Table 2.** Effects of different prepared nanoformulations (CS NPs, Cur@CS NPs, and Amp/Cur@CS NPs) on serum immunity indices in rat models.

Item	Control	CS NPs	Cur@CS NPs	Amp/Cur@CS NPs
IgG (g/L)	1.10	1.25	1.34	1.36
IgM (g/L)	2.30	2.39	2.40	2.48
IgA (g/L)	0.806	0.565	0.530	0.643
C3	0.040	0.039	0.038	0.041
C4	0.035	0.039	0.040	0.038

### 3.8. *In vivo* toxicity analysis

**Figure 11.**
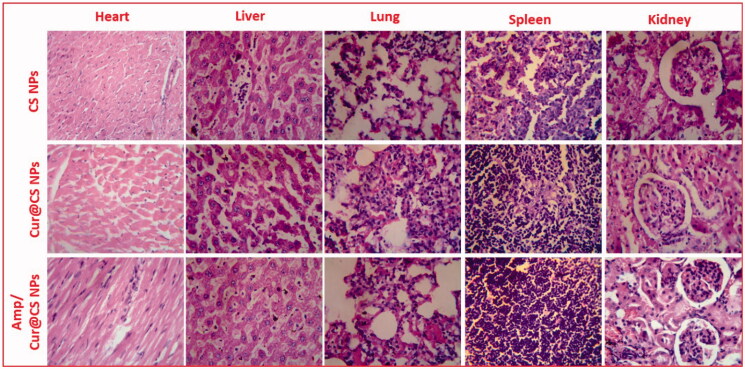
Histomorphology photographs of *in vivo* toxicity of treated samples (CS NPs, Cur@CS NPs, and Cur/Amp@CS NPs) on major organs (heart, liver, lung, spleen, and kidney) of the rat model.

It is very essential to note that prepared nanoformulations would circulate in whole blood in the body and may influence functions of major organs (i.e. heart, lungs, liver, spleen, and kidneys); hence, it should be investigated through *in vivo* biosafety analysis. Generally, histopathological (H&E staining) visualization is an extensively applied method and was performed to evaluate structural damages on the major organs as exhibited in Figure 11. The observed results demonstrated that no histomorphological modifications were observed in the organs after IV injection of developed nanoformulations *in vivo*, which revealed that prepared carrier materials have excellent biosafety behavior for biomedical applications. Importantly, organs pathological observations were displayed with normal cellular and tissue morphology without any damaged organ structure.

## 4. Conclusions

In summary, we established that developed CS nanoformulation loaded with Cur and AMP molecules can successfully inhibit bacterial infections without disturbing intestinal microflora when compared to the commercial antibiotics. The outstanding cytocompatibility and efficient bacterial inhibition of Cur/Amp@CS nanoformulations were proved by using intestinal MODE-K cells and *E. coli* bacterial cells. The Cur/Amp@CS nanoformulation has significantly augmented the RA of intestinal probiotics, and immunity without disturbing α-diversity could substantially protect the rat intestinal microflora. *In vivo* biosafety histomorphological results confirmed that nanomaterials have no toxicity to major organs of rat models by IV injection. The developed Cur/Amp@CS NPs have effectively balanced intestinal microflora with inhibition of bacterial cells, which could be used as considerable substitute to antibiotics for intestinal infection-based therapies.

## Supplementary Material

Supplemental Material
