# Supplementary material for: Fabrication of bio-engineered chitosan nanoformulations to inhibition of bacterial infection and to improve therapeutic potential of intestinal microflora, intestinal morphology, and immune response in infection induced rat model
Source: Drug Deliv. 2022 Jun 29;29(1):2002–16. doi: 10.1080/10717544.2022.2081381 (PMC9255213; doi:10.1080/10717544.2022.2081381)
Supplement: Supplemental Material [file IDRD_A_2081381_SM8973.docx]

**Supplementary Information**

**Fabrication of Bio-engineered Chitosan Nanoformulations to inhibition of bacterial infection and to improve therapeutic potential of intestinal microflora, intestinal morphology and immune response in infection induced Rat model**

**Xiao Wan^1^, Liu Liu^1^, Lu Ding^2^ and Zhiqiang Zhu^1,*^**


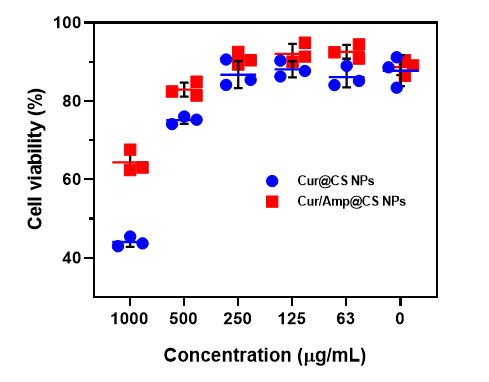


**SI 1.** *In vitro* quantitative analysis of cell viability of MODE-K cells with different concentrations of prepared samples.

**SI 2.** Measurement of Intestinal microflora F/B value after treatment of different concentrations of Cur@CS NPs and Cur/Amp@CS NPs for 28-days of oral administration.


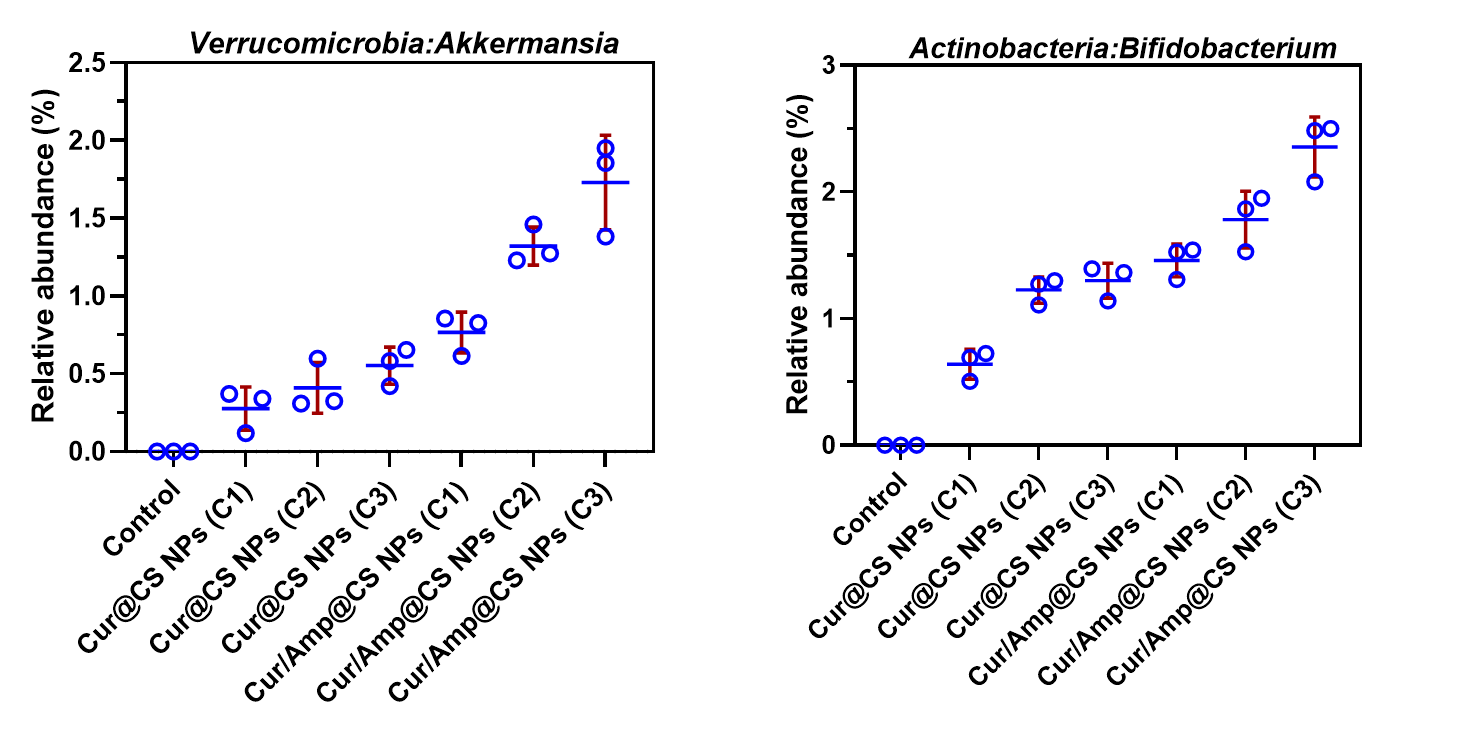


**SI 2.** Relative abundance of genera in microflora after the oral 28-days oral administration of Cur@CS NPs and Cur/Amp@CS NPs.
